# Climate change and infectious disease: a review of evidence and research trends

**DOI:** 10.1186/s40249-023-01102-2

**Published:** 2023-05-16

**Authors:** Paige Van de Vuurst, Luis E. Escobar

**Affiliations:** 1 Virginia Tech Graduate School, Translational Biology, Medicine, and Health Program, Blacksburg, VA USA; 2 Department of Fish and Wildlife Conservation, Virginia Tech, Blacksburg, VA USA; 3 Center for Emerging Zoonotic and Arthropod-Borne Pathogens, Virginia Tech, Blacksburg, VA USA; 4 Global Change Center, Virginia Tech, Blacksburg, VA USA; 5 Facultad de Ciencias Agropecuarias, Universidad de La Salle, Bogotá, Colombia

**Keywords:** Climate change, Infectious disease, Research trend, Systematic review

## Abstract

**Background:**

Climate change presents an imminent threat to almost all biological systems across the globe. In recent years there have been a series of studies showing how changes in climate can impact infectious disease transmission. Many of these publications focus on simulations based on in silico data, shadowing empirical research based on field and laboratory data. A synthesis work of empirical climate change and infectious disease research is still lacking.

**Methods:**

We conducted a systemic review of research from 2015 to 2020 period on climate change and infectious diseases to identify major trends and current gaps of research. Literature was sourced from Web of Science and PubMed literary repositories using a key word search, and was reviewed using a delineated inclusion criteria by a team of reviewers.

**Results:**

Our review revealed that both taxonomic and geographic biases are present in climate and infectious disease research, specifically with regard to types of disease transmission and localities studied. Empirical investigations on vector-borne diseases associated with mosquitoes comprised the majority of research on the climate change and infectious disease literature. Furthermore, demographic trends in the institutions and individuals published revealed research bias towards research conducted across temperate, high-income countries. We also identified key trends in funding sources for most resent literature and a discrepancy in the gender identities of publishing authors which may reflect current systemic inequities in the scientific field.

**Conclusions:**

Future research lines on climate change and infectious diseases should considered diseases of direct transmission (non-vector-borne) and more research effort in the tropics. Inclusion of local research in low- and middle-income countries was generally neglected. Research on climate change and infectious disease has failed to be socially inclusive, geographically balanced, and broad in terms of the disease systems studied, limiting our capacities to better understand the actual effects of climate change on health.

**Graphical abstract:**

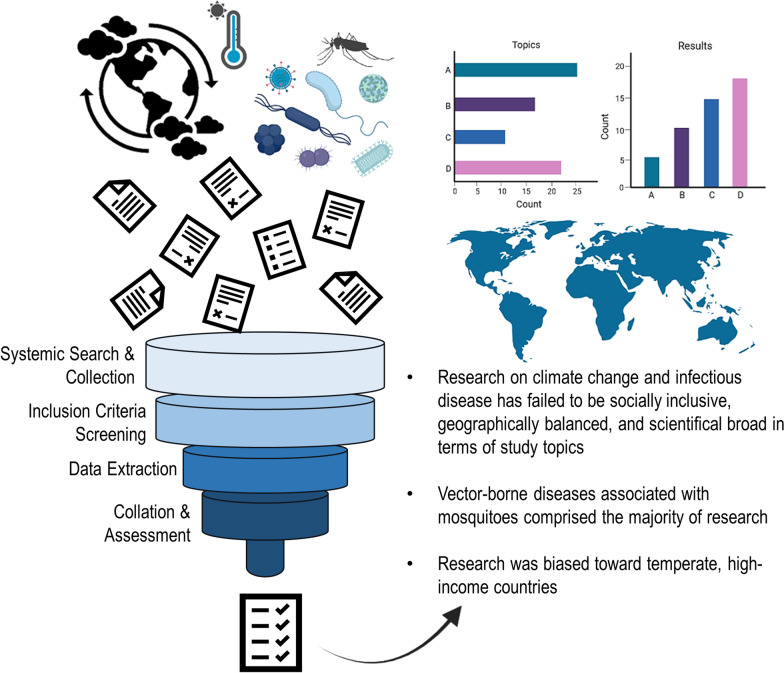

**Supplementary Information:**

The online version contains supplementary material available at 10.1186/s40249-023-01102-2.

## Background

The Intergovernmental Panel on Climate Change has anticipated, with high confidence, that climate change will amplify health threats worldwide [1, 2], which is supported by the fact that the life cycles of many infectious agents are inextricably linked to climate [1, 3–6]. Multiple studies have shown that variation in temperature, precipitation, and humidity affects the transmission and distribution of infectious diseases [7–10]. Nevertheless, the magnitude, direction, and strength of the impact of climate change upon infectious disease transmission remains unclear [3, 5, 7]. To determine what further research is needed to advance a given field in scientific research it is often necessary to synthesize previous work [11]. This type of retrospective, systematic analysis of literature in a specific topic or field is referred to as a systematic review. Systematic reviews are a popular and effective method commonly utilized to identify trends and gaps in ongoing research [12]. Results from systematic reviews and scoping studies, which are often used to map the availability of literature on an specific topic [13, 14], can be used to guide future research lines, future policy decisions, and can be particularly useful in scientific fields with emerging evidences, such as epidemiology [12, 13, 15, 16].

Despite their effectiveness, systematic reviews are noticeably lacking in the literary landscape of anthropogenic climate change research, especially with regard to its impacts on infectious diseases. There is, therefore, a need for a systematic synthesis of recent empirical research assessing disease impacts of climate change. Here, we provide a synthesis of scientific literature on climate change and infectious diseases from recent history. The overall objective of this study was to determine the trends of recent empirical research regarding climate change impacts on infectious diseases and to identify geographic, topical, or taxonomic trends of research. We sought to assess the geographic regions where climate change and disease transmission have been under studied, accounting for both study area and first author affiliation to identify geographic and bibliometric signals. In addition, we assessed the taxa of hosts and transmission types of pathogens studied. Finally, we sought to inform future research avenues, policy, and practices via the trends and impacts identified herein.

## Methods

### Search strategy, inclusion criteria, exclusion criteria

Our search strategy included recovering articles from Web of Science (Clarivate™) [17] and PubMed™ [18] literary repositories using a key word search. Keywords included "climate change", "global warming", “greenhouse gas*” (*asterisk used to incorporate all forms of the word. i.e., gas, gases, gaseous), “world warming”, “disease”, “infectious”, “pathogen”, “waterborne”, “water borne”, “food borne”, “vector borne”, “parasite”, and “non-vector borne”. Time range restrictions were set from January of 2015 to December of 2020 to incorporate all publications from the most recent, pre-pandemic five-year period of empirical climate change research. This key word search was limited to journal manuscripts, as the purpose of this study was to analyze original peer-reviewed research. Other literature types such as book chapters, review articles, proceedings papers, or conference abstracts were excluded. Articles were then imported into Endnote citation software, where redundant articles were removed.

After collection we conducted an initial screening of both article titles and abstracts. This initial review allowed for the identification of articles which did not fit within the review criteria. Inclusion criteria were: (1) The manuscript was peer-reviewed and published without retraction, (2) the primary goal of the research was centered on assessing climate change and its repercussions, impacts, effects, association, or influences on disease, infection, transmission, infestation, or illness, (3) the research was original and not a review, (4) the research was descriptive, retrospective, and based on real world systems using non-simulated future-climate data (i.e., present-day and past climate only), (5) the manuscript utilized primary data and (6) the pathogen, parasite, vector, or disease of focus impacted either humans, non-human animals, or both. Each article was reviewed by at least two independent reviewers and was confirmed for inclusion or exclusion based on the inclusion criteria. If the independent reviewers were in disagreement on whether or not the article fit the inclusion criteria, the article was reviewed by a third reviewer. Studies which did not fit this inclusion criteria were flagged and maintained in a separate databased. Studies on plant diseases were not within the scope of this study and therefore were excluded.

### Evidence extraction and analysis

We then reviewed the remaining publications in full and conducted evidence extraction of each article to conduct our gap analysis of bibliometric, subject, taxonomic, and geographic trends in research and publication. We gathered descriptive metadata from each article to assess when, where, and by whom the articles were published (e.g., year or publication, journal name, title, authors, etc.). To assess authorship demographics, we recorded the lead author and senior author’s names, pronouns, and institutional affiliation for each publication. Authors’ pronouns were recorded based upon the personal distinctions of each individual author, and the pronouns they chose to use (e.g., she/her, he/him, they/them, em/eir, xem/xyr, etc.) on their institutional or research affiliated websites. We implemented this method to be inclusive of all authors’ identities while maintaining personal privacy [19, 20]. If the author did not denote their pronouns in any public way, we recorded their pronouns as “unknown”. We also collected descriptive metadata on the study methods and locations or each article including: (1) study location at the country and continent level, (2) disease host, vector, or pathogen studied, (3) transmission method of each disease studied, (4) primary taxa or taxon of interest (i.e., the taxonomic group of the host or infectious organism or organisms being studied), and (5) spatial scale (e.g., local or inferior to country level, regional, country level, or global). To assess the quality of the included literature, we also recorded and synthesized the conclusions of the sampled articles, and reported these findings based upon the author’s interpretation of their results. We also collected descriptive information on the publication funding or support for each article published in the most recent year included in the review (i.e., 2020) to ascertain current funding sources for the most recent climate change and disease publications. We then compared funding sources with current estimates of country gross domestic product (GDP) from the World Bank World Development Indicators Dataset [21].

To assess the distribution of the categorical topics of the literature we used a Pearson’s chi-squared (*χ*^2^) test. It has been estimated that approximately 60% of known infectious diseases are zoonotic (i.e., originating in non-human animals) [16, 22]. We compared this value (60%) with the proportion of literature which assessed zoonotic diseases to identify if the literature followed this expected proportion. We also used the *χ*^2^ test to identify if the proportion of host species categories studied (humans, wildlife, and livestock) were equal. To assess the geographic distribution of publication demographics, the lead authors’ institutional affiliations were recorded for each publication and assigned to their corresponding countries of origin. Demographic data of study locations and author affiliations were summarized and visualized to detect spatial and temporal patterns of these data using ArcGISpro version 2.9.3 and R version 4.1 [23–25]. We utilized population data from the United Nations Population Division [26] for the year 2020 to assess the per-capita research effort by country.

## Results

### Literature demographics

Our initial key word search resulted in 10,461 articles from both PubMed and Web of Science. A total of 621 research articles (5.9%) fit the inclusion criteria for the 2015–2020 period and were retained for evidence extraction and gap analysis. Within these publications, 109 distinct infectious diseases were identified in relation to climate change research. A small portion of publications (*n* = 127) assessed multiple diseases within the same study. Authors of the reviewed articles reported that climate change impacted the disease system being assessed in 59% of the articles. Most of the articles (83.9%) which described climate change impacts reported that climate change increased the prevalence, transmission, or suitability for the disease being studied, while 11.5% of studies reported that climate change decreased the prevalence, transmission, or suitability. Only 7.7% of the assessed articles reported no effect of climate change on the disease system being studied. The review revealed that 32.7% of the articles concluded that climate change could “possibly” or “potentially” impact the disease system being assessed (i.e., the authors did not report a definitive pattern).

### Research trends

Infectious diseases which originate from cross-species pathogen transmission of animals to humans (i.e., zoonotic diseases) accounted for most of the studies (*n* = 288, 46.4%), significantly more than diseases which do not originate from animal to human cross species transmission (*n* = 253, 40.7%), (*χ*^2^ = 9.97, *P* = 0.002). Infectious diseases which impact humans were well represented within the literature (*n* = 406) (*χ*^2^ = 114.3, *P* = 0.0001), while infectious diseases affecting livestock were less represented (*n* = 152). Only 116 publications assessed diseases affecting wildlife.

**Fig. 1:**
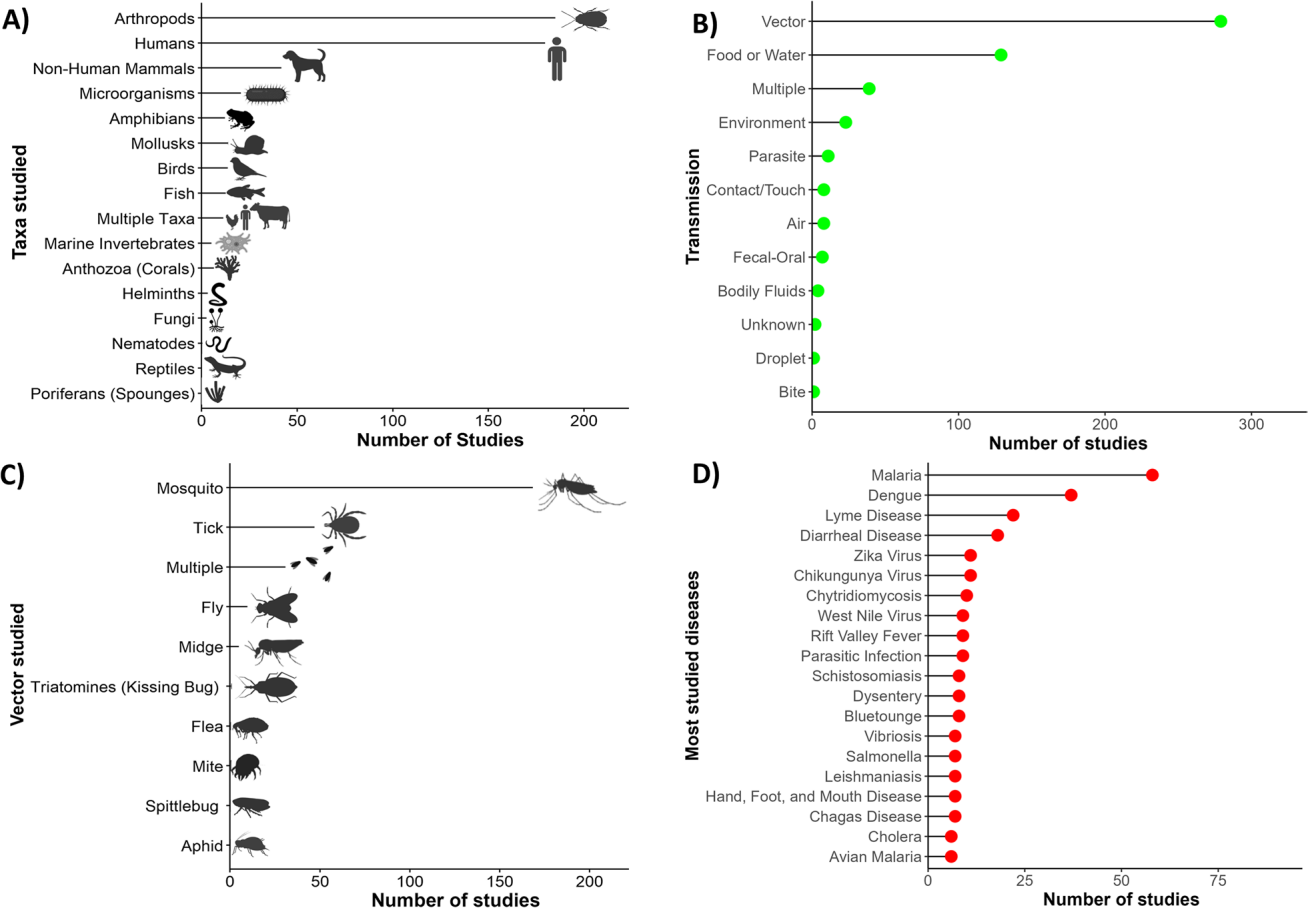
Trends in climate change and disease research. Number of publications (*x*-axis) from 2015–2020 according to **A** taxa of host species studied, **B** transmission type of diseases studied, **C** vector species studied, and **D** top 20 most studied diseases from over 100 different diseases studied. Multiple: multiple diseases with multiple transmission types studied in a single article

**Fig. 2:**
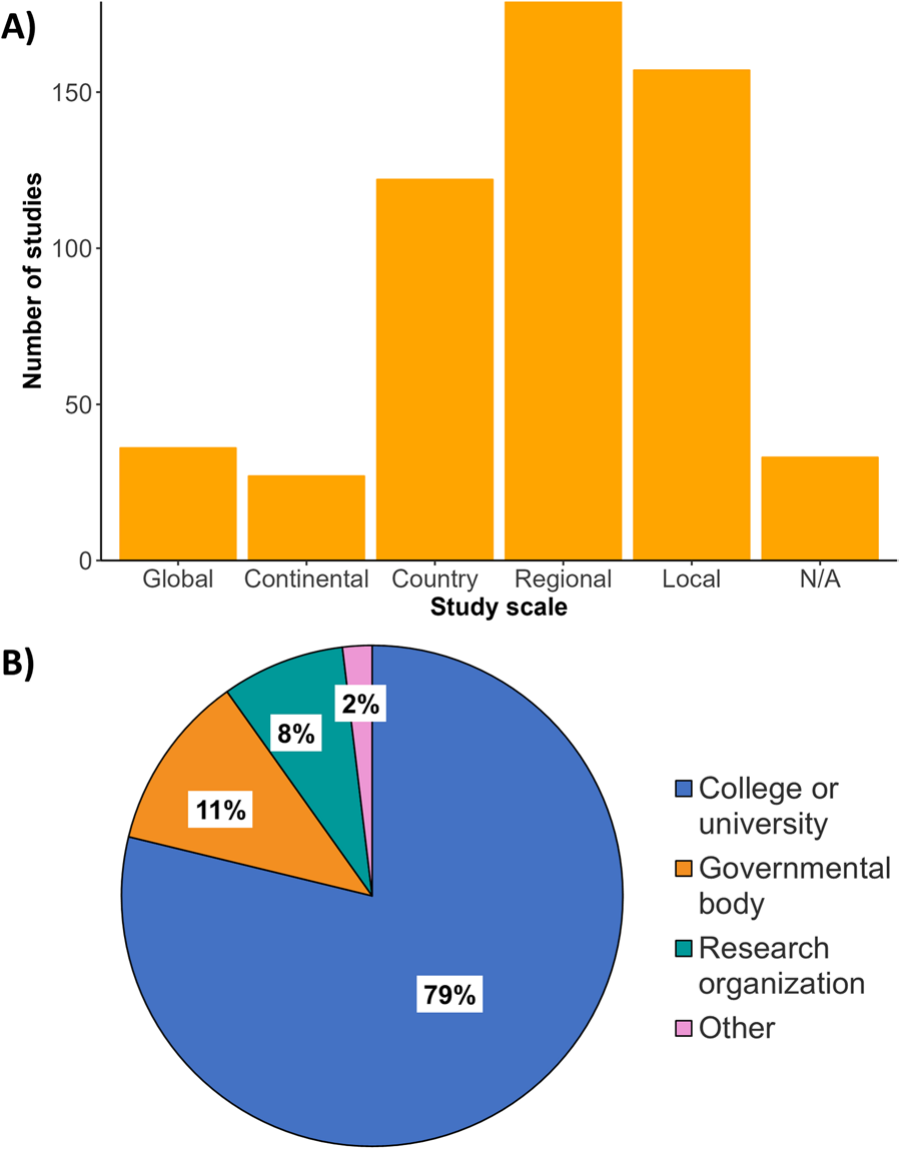
Bibliometric demographics. **A** Number of publications (*x*-axis) from 2015–2020 when delimited by scale of study. N/A: Studies for which a spatial scale was not applicable (e.g., laboratory-based studies) or for which scale was not specified. **B** Percentile breakdown of lead author affiliations collated into categories based on the institution’s description (i.e., college or university, governmental organizations or research organization). Other: lead author affiliation institutions which do not fit one of these categories including non-governmental organizations, independent researchers, or private companies not otherwise specified

The specific conditions most frequently studied from this sample included vector-borne diseases (Fig. 1), such as malaria (*n* = 58), dengue fever (*n* = 37), and Lyme disease (*n* = 22) (Fig. 1). Vectors most frequently studied were mosquitoes (*n* = 174), ticks (*n* = 51), and flies (*n* = 14) (Fig. 1). Frequently studied environmentally transmitted conditions included food and water-borne diseases, such as diarrheal diseases (*n* = 18) and chytridiomycosis (*n* = 10) (Fig. 1). Studies also focused on diseases hosted by arthropods (*n* = 189) and humans (*n* = 185) (Fig. 1). The third most studied host taxonomic group was non-human mammals (*n* = 47), followed by amphibians (*n* = 19) and birds (*n* = 17) (Fig. 1). In terms of study scale, research was conducted at the local, regional, or country levels, with less effort for global-level studies (Fig. 2).

### Publication trends

**Fig. 3:**
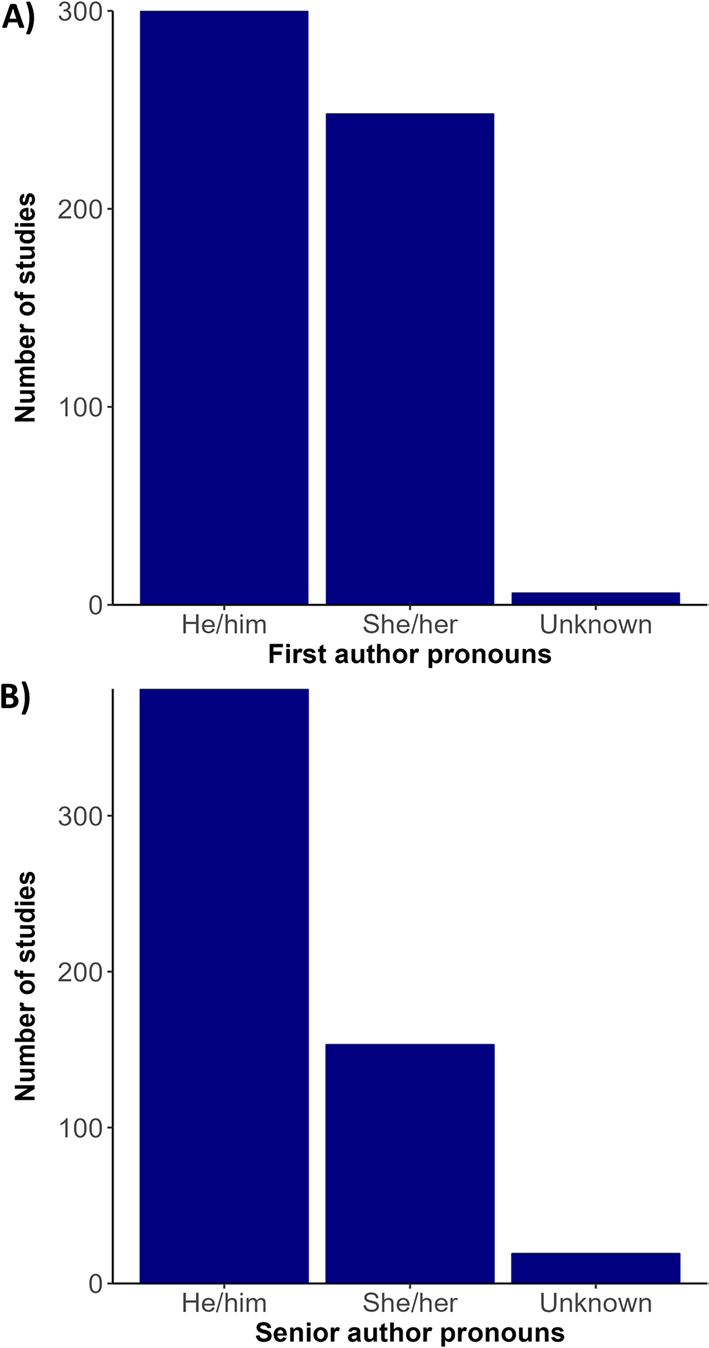
Author pronouns on climate change and infectious disease research. The self-identified pronouns of **A** first authors and **B** last (senior) authors of articles on climate change and disease from 2015 to 2020. The disparity between he/him pronoun usage over other pronouns was pronounced for senior authors. Authors’ pronoun usage in public settings may vary from their gender identities

**Fig. 4:**
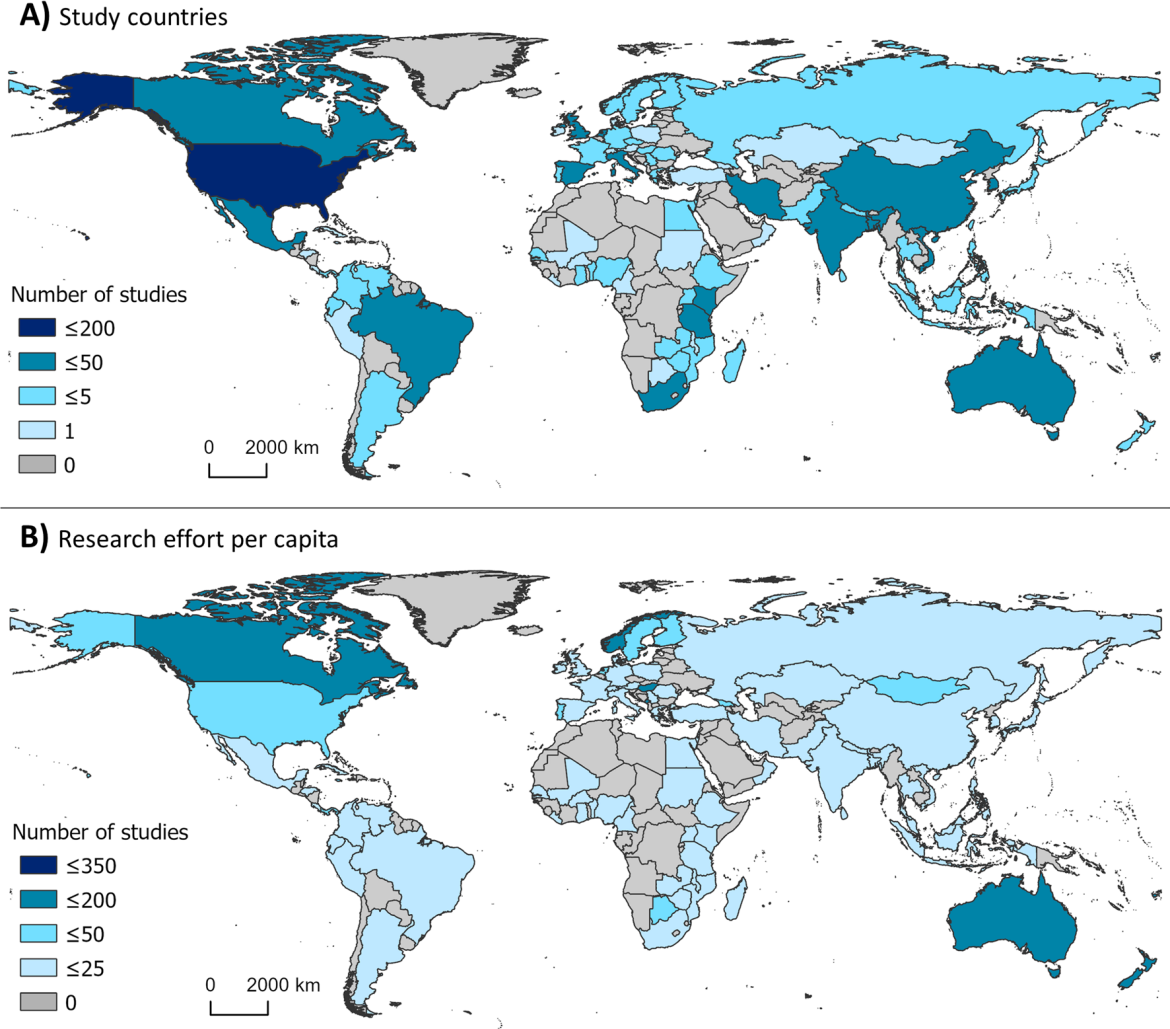
Map of study locations by country. **A** The geographic representation of where studies were conducted (i.e., country where the data analyzed in the study originated) from 2015–2020 on climate change and infectious disease and **B** publications that fit the inclusion criteria as a proportion of human population in 2020 (per one million individuals). Population data were collected from the United Nations Population Division [26]. Darker color represents more publications conducted in or on the corresponding country. Grey indicates that no studies which fit the inclusion criteria were conducted in or on the corresponding countries. Shape file for map creation sourced from DIVA-GIS [84, 85]

**Fig. 5:**
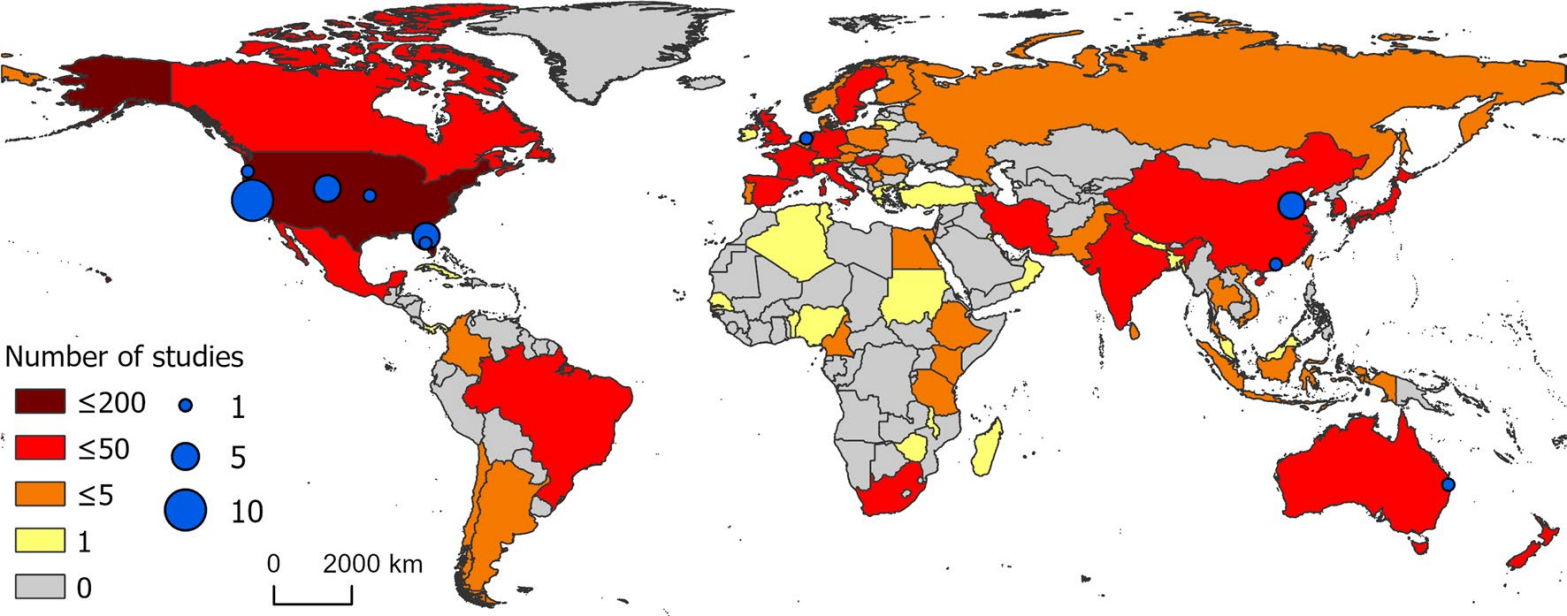
Map of lead author affiliation origins. The geographic representation of lead author affiliation origins for research on climate change and disease from 2015 to 2020. Darker color represents more publications originating from the corresponding country. Grey indicates that no studies which fit the inclusion criteria were conducted by authors affiliated with the corresponding countries. Blue points indicate the top ten publishing institutions globally for climate change and disease. Shape file for map creation sourced from DIVA-GIS [84, 85]

Bibliometric analysis revealed a greater usage of he/him pronouns for both first and senior authors (Fig. 3). We recorded no instances of they/them or other non-binary pronouns by first or senior authors from the articles revised. We also found that study areas and affiliation of lead authors most frequently occurred in the United States, China, the United Kingdom, Canada, and Australia (Figs. 4, 5). Research effort accounting for the country’s population size showed that countries such as Norway, Australia, and Canada have a higher comparative research effort than other countries (Fig. 4). Most lead author affiliations were linked to higher education institutions (i.e., universities or colleges), with fewer publications originating from governmental organizations or independent research institutions (Fig. 2). University affiliations were frequently located in the United States (e.g., the University of California, Colorado State University, University of Florida), and in China (e.g., Shandong University) (Fig. 5). Funding for papers published in 2020 was largely sourced from federal or national institutions (53.3% of articles) or a combination of federal and academic institutions (26.7% of articles), with most of this funding originating in high income countries such as the United States, Canada, Germany, and the United Kingdom (Supplementary Fig. 1). Information of funding sources from lower income countries was limited, with only one country (Greece) having a GDP below the top 50 of reported counties based on World Bank estimates [21]. Non-governmental organizations and local agencies made up a modest proportion of funding sources for the total of articles published (20%).

## Discussion

Through this study we have revised the major trends in the current literature on climate change and infectious diseases. Our assessment identified both topical and geographic biases in the climate change and disease research arena. More specifically, we found that there was a notable focus on diseases which impact humans and upon arthropod-borne pathogens. Taxonomic bias, or the emphasis of study on specific organisms [27], has previously been identified in biodiversity and conservation science research [28–30]. Our results have identified taxonomic biases toward mammalian hosts and arthropod-borne pathogens and in climate change and infectious disease research. When certain taxa are over-represented in various scientific fields it is possible for them to draw both attention and funds away from less understood taxa [28]. It is possible that taxonomic bias has impacted the study of climate change and infectious disease by skewing research toward specific disease systems, suggesting an anthropocentric research approach potentially influenced by external forces, such as public health funding and disease burden [31, 32]. Vector-borne diseases have considerable burden on human health, killing approximately 700,000 people annually [33]. A research emphasis on diseases affecting humans is, therefore, potentially unsurprising as human health is a driving force behind many research efforts and encompasses a large proportion of research and development funding [34, 35]. Other research has shown that societal pressures correlate with taxonomic bias [28], which could explain why human-only and zoonotic diseases were so heavily studied as well.

Despite the anthropocentric nature of our results, many understudied taxa, such as amphibians, birds, and aquatic invertebrates, have higher risks of extinction due to infectious diseases than humans or other mammals [36–38]. Taxonomic bias in the study of infectious disease is concerning, as a lack of research effort could limit the understanding of diseases systems for threatened or endangered taxa. This in turn limits our capacities to understand how, where, and why diseases emerge in the wild. Risks of climate change impacts on lesser studied groups, such as wildlife and livestock, could still have public health effects due to spillover transmission of unknown pathogens [22, 39]. The dearth of research on wildlife diseases could also lead to gaps of knowledge. Infectious diseases may harm ecological balance by reducing wildlife populations and decreasing overall biodiversity [40–42]. A large body of literature shows that ecological imbalances and biodiversity loss have detrimental effects on human health as well [39, 43–45]. For instance, decreases in diversity of wildlife has been associated with increases risk of hantavirus spillover transmission from rodents to humans [46–49]. Public health efforts to study climate change and human health should consider biodiversity dimensions of spillover transmission for a more holistic ecosystem health approach.

We found that most lead authors were linked to higher-education institutions (i.e., universities or colleges), with fewer publications originating from governmental organizations or independent research institutions (Fig. 2). This bias towards academic-based research is not surprising considering that higher-education institutions often focus efforts on research and disseminating knowledge [50]. This result also indicates a poor active participation of stakeholders in governing bodies on climate change and health research, which could explain the slow progress of international policy on climate change and disease research. It is important to note, however, that most funding for the support of recent research publications originated from federal or national institutions (Additional file 1: Fig. S1). While funding agencies constitute important stakeholders in the scientific publication process, agendas from funding sources may bias the research topics and discoveries reported [51, 52]. For instance, publications with corporate funding are more likely to contribute to the polarization or politicization (i.e., contributing to the tension between political ideologies or identities) of climate change related topics [53]. We found that most articles reviewed for funding sources did not receive funding from corporate or industry agencies. Government funding is the main driver of science and provides research directions for non-government funding sources [52]. As such, an increase in government funding for climate change and infectious disease research accounting for environmental justice could transform the landscape of public and private research funding opportunities to reduce the inequities presented here. An increase in funding in the social science aspects of climate change may also facilitate the framing of climate change as a global social challenge, rather than a purely scientific endeavor with limited social legitimacy [54].

We also found that there was greater usage of he/him pronouns by lead and senior authors across the articles revised, suggesting that more male or male identified authors were present than female or female identified authors (Fig. 3). Gender discrepancies in authorship were more notable for senior authorship than for first authorship, which appears to be a general pattern in academic authorship inequity [55], even with increased authorship by women in recent decades [56]. Until recently, women or female-identified authors comprise a minority of researchers and trainees in science in general, which has resulted in authorship inequities that are expected to persist for some time [56]. Gender persistant inequity in authorship is specifically conerning within the field of climate change and infectious disease research due to its cross cutting social implications. Women are expected to experience greater climate change and health impacts as a result of their social and economic positions, and cultural discrimination [57]. As such it is important that women’s viewpoints and experiences are represented within the scientific literature to develop more effective and inclusive policies for climate change adaptation and mitigation.

In terms of geographic scale and location, we found that most climate change and infectious disease research was conducted at the regional and local scales (Fig. 2), suggesting that fine-scale studies dominate the field and our understanding of climate change impacts on human and animal health. Climate change and disease research also occurred principally in temperate areas (e.g., North America, Europe) rather than in tropical areas (e.g., sub-Saharan Africa, Latin America, and Pacific Southeast Asia) (Figs. 4, 5). This spatial bias is present even when publications were corrected for country population. The research effort discrepancy between temperate vs tropical regions is concerning considering that tropical areas are the most at risk for emerging infectious diseases impacts [58, 59]. Tropical areas are also experiencing drastic climate change effects, including reductions in food availability in short periods [60]. Tropical areas having limited to no climate change and disease research included Latin America, Northern and West Africa, and the Indo-pacific (Figs. 3, 4). Furthermore, climate change is expected to increase the areas suitable for infectious agents in land and aquatic ecosystems [10, 61]. For instance, the aquatic pathogen *Vibrio cholerae*, the causative agent of cholera, is expected to increase in regions where we found limited research effort [61, 62]. Other areas which did not receive substantive research effort include extremely cold Arctic or Subarctic areas of Eurasia (Fig. 4). Permafrost regions such as these have recently experienced outbreaks of avian influenza (H5N1) [63], and previous reviews have identified melting permafrost as a reservoir of potentially viable and uncharacterized pathogens [64]. As such, a constituted effort to elucidated emerging infectious diseases in these regions should be undertaken to mitigate the risks of disease emergence. The confluence of susceptibility to both climate change impacts and infectious disease suggests a need for research in underrepresented areas reported here. Furthermore, underrepresentation of countries and human communities already disenfranchised and at greater risk for encountering infectious disease amplifies social inequity [7].

One caveat of our assessment is that publications from lower income or developing countries may not have been indexed in the publication data repositories accessed (i.e., Web of Science and PubMed) due to publication barriers such as language, publication fees, or lack of equitable partnerships or collaborative networks [65–69]. The potential misrepresentation of science from low-income countries highlights a possible equity issues within the dissemination of research which, in turn, could lead to the exclusion of relevant discoveries in the global health agenda [68–70]. A confirmation or publication bias could also be present in our results, as seen by the high number of papers which positively identified a climate change impact on infectious diseases. Previous research has commented on the scientific culture and potential dangers associated with the current emphasis on publishing only “significant” or “positive” results [71–74]. It is possible that researchers were reluctant or unable to publish negative or inconclusive results, thus skewing the conclusions of this sample. Furthermore, while we found that many articles either found a definitive climate change impact, or concluded that climate change could “possibly” or “potentially” impact the disease system being assessed, these findings were based upon the author’s interpretation of their results and may be an exaggerated interpretation of the data. Finally, while we sought to identify the distribution of authorship via author pronoun usage, there could be discrepancies present between the pronouns publicly available for the authors and the gender identities they have privately. This discrepancy is to be expected considering the discriminatory practices in academia against lesbian, gay, bisexual, transgender, and queer (LGBTQ+) scientists [75–77].

## Conclusions

We found that both geographic and taxonomic trends were present in recent studies assessing climate change and the burden of infectious disease. The majority of research was focused on vector-borne pathogens and was conducted in well-developed, high-income countries with temperate climates, neglecting directly-transmitted diseases in tropical regions. The anthropocentric signal in research effort may contribute to a lack of understanding of climate change effects on wildlife systems. The underrepresentation of some taxonomic groups of pathogens and hosts, pathogen transmission types, and geographic areas should be of global health concern, as areas and diseases neglected may become sources of emerging zoonotic diseases. An ecosystem-based framework to study disease responses to climate change could mitigate topical and taxonomic biases identified here. Viral zoonoses outbreaks at the local level in underrepresented countries such as Madagascar, Saudi Arabia, and Indonesia have led to prolific human epidemics of plague, Middle East respiratory syndrome, and cholera in recent years [78], highlighting the need for more research in regions underrepresented in the literature. The recent coronavirus disease pandemic also highlights the need for more research on directly transmitted pathogens circulating in wildlife [79]. Furthermore, research is still needed to understand the linkages between patterns of research funding with climate change and infectious disease studies. Understanding the funding landscape (e.g., agencies prioritizing certain regions, diseases, and topics) could further elucidate the relationship between research bias, research equity, and funding allocation.

The impact of climate change research on intergovernmental policy and vice versa is both tractable and increasingly important [80, 81]. Policy changes to address the biases presented here, including the diseases studied, areas, and identities of leading authors, should be prioritized by both funding agencies and the scientific community. Policy change could include, for example, the prioritization of infectious disease research and surveillance at the human-wildlife interface within the context of climate change, funding prioritizing scientists from minority groups, and neglected geographic regions. Addressing research inequity will help build human capacity, surveillance, and scientific infrastructure to better prepare and strengthen the global health response to climate change threats [82]. Furthermore, research foundations in high-income countries should implement and maintain inclusive-collaboration practices to value contributions by local scientists in countries underrepresented in this review to advance research equity as a means towards effective prevention of future emerging diseases from their sources. Building political and social support behind climate change and infectious disease research will be essential under the expected rates of climatic variation in the near future [83]. In conclusion, there is an urgent need to increase research effort for neglected disease systems and geographies, and there is a need to re-examine aspects of environmental justice from the scientists leading these studies to the local beneficiaries for the advancement of infectious diseases research in the context of climate change.

## Supplementary Information

**Additional file 1: Figure S1.** Sources of publication funding or support from articles published in 2020. For all articles which fit the inclusion criteria that were published in the year 2020 we extracted the funding or support source listed in the article. These funding sources were classified as federal, non-governmental organizations such as charities or independent research organizations, local agencies, or a combination of federal and academic or federal and industry support.

## Data Availability

Not applicable.

## Abbreviation

GDP: Gross domestic product
