# Supplementary figures and images for: Climate change and infectious disease: a review of evidence and research trends
Source: Infect Dis Poverty. 2023 May 16;12:51. doi: 10.1186/s40249-023-01102-2 (PMC10186327; doi:10.1186/s40249-023-01102-2)

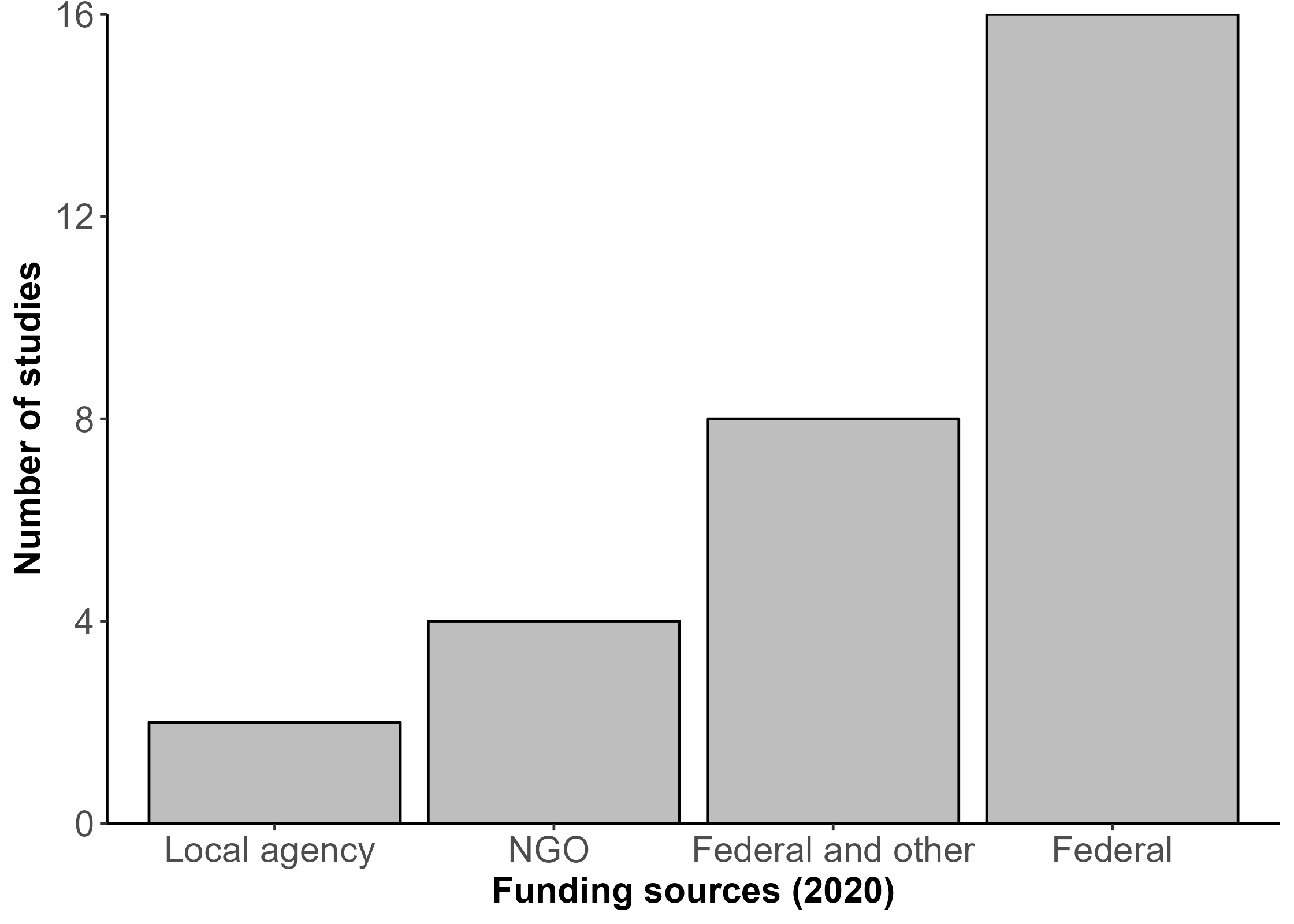

Supplement: Supplementary file 1 — Additional file 1: Figure S1. Sources of publication funding or support from articles published in 2020. For all articles which fit the inclusion criteria that were published in the year 2020 we extracted the funding or support source listed in the article. These funding sources were classified as federal, non-governmental organizations such as charities or independent research organizations, local agencies, or a combination of federal and academic or federal and industry support. [file 40249_2023_1102_MOESM1_ESM.tif]
